# Supplementary material for: What determines client satisfaction on labor and delivery service in Ethiopia? systematic review and meta-analysis
Source: PLoS One. 2021 Apr 22;16(4):e0249995. doi: 10.1371/journal.pone.0249995 (PMC8061977; doi:10.1371/journal.pone.0249995)
Supplement: S2 File — (DOCX) [file pone.0249995.s002.docx]

**Sample search string for CINHAL database, EBSCOhost Interface**

| **#** | **Query** | **Limiters/Expanders** | **Last Run Via** | **Results** |
| --- | --- | --- | --- | --- |
| S4 | (Ethiopia) AND (S1 AND S2 AND S3) | Search modes - Find all my search terms | Interface - EBSCOhost Research Databases  Search Screen - Advanced Search  Database - CINAHL Complete | 11 |
| S3 | Ethiopia | Search modes - Find all my search terms | Interface - EBSCOhost Research Databases  Search Screen - Advanced Search  Database - CINAHL Complete | 1,043 |
| S2 | Client satisfaction/women satisfaction | Search modes - Find all my search terms | Interface - EBSCOhost Research Databases  Search Screen - Advanced Search  Database - CINAHL Complete | 2508 |
| S1 | factors associated OR Determinants OR Predictors | Search modes - Find all my search terms | Interface - EBSCOhost Research Databases  Search Screen - Advanced Search  Database - CINAHL Complete | 11480 |

**Sample search string for Medline database, EBSCO host Interface**

| **#** | **Query** | **Limiters/Expanders** | **Last Run Via** | **Results** |
| --- | --- | --- | --- | --- |
| S4 | (Ethiopia) AND (S1 AND S2 AND S3) | Search modes - Find all my search terms | Interface - EBSCOhost Research Databases  Search Screen - Advanced Search  Database - MEDLINE | 62 |
| S3 | Ethiopia | Search modes - Find all my search terms | Interface - EBSCOhost Research Databases  Search Screen - Advanced Search  Database - MEDLINE | 9097 |
| S2 | Client satisfaction/women satisfaction | Search modes - Find all my search terms | Interface - EBSCOhost Research Databases  Search Screen - Advanced Search  Database - MEDLINE | 127 |
| S1 | factors associated OR Determinants OR Predicators | Search modes - Find all my search terms | Interface - EBSCOhost Research Databases  Search Screen - Advanced Search  Database - MEDLINE | 13911 |
