# Supplementary material for: What determines client satisfaction on labor and delivery service in Ethiopia? systematic review and meta-analysis
Source: PLoS One. 2021 Apr 22;16(4):e0249995. doi: 10.1371/journal.pone.0249995 (PMC8061977; doi:10.1371/journal.pone.0249995)
Supplement: Attachment — Submitted filename: PONE-D-20-11487- OBj - Client Satisfaction on labour and delivery services.pdf [file pone.0249995.s005.pdf]

PLOS ONE

What determines client satisfaction on labor and delivery service in Ethiopia? systematic review and meta-analysis

--Manuscript Draft--

Manuscript Number:	PONE-D-20-11487
Article Type:	Research Article
Full Title:	What determines client satisfaction on labor and delivery service in Ethiopia? systematic review and meta-analysis
Short Title:	Client satsifaction on labor and delivery service
Corresponding Author:	REKIKU FIKRE ABEBE, BSc in midwiverey<br>Hawassa University<br>hawassa, ETHIOPIA
Keywords:	client satsifaction, labour and delivery, Meta-analysis
Abstract:	Abstract<br>Introduction: The uptake of service utilization increments dictated by the satisfaction of clients and the outcome of mother and newborn. Client satisfaction is one of the vital pointers to measure the quality of servce based up on satisfaction of the client. However, it is underwhelmed and little evidence is existed in Ethiopia .Therefore, the purpose of this systematic review aimed to assess the prevalence and determinant of client satisfaction on labor and delivery service in Ethiopia.<br>Methodology : This studies were included published and unpublished articles. The main databases PubMed, Embase, EBSCO, Medline, CINHAL, Poplin, and the search engine like google and google scholar were used. Observational study design studies were conducted in the English language and met the eligibility criteria were included in the review. Meta-analyses with random effects were performed. Data synthesis and statistical analysis were conducted using OpenMeta and CMA version 2 software.<br>Results: The pooled prevalence of client satisfaction on labor and delivery service in Ethiopia was73.5% [95%CI [64.9%,82.1%]. The pooled odds ratio showed that a positive association between client satisfaction on labour and delivery service with Promptness of care [OR, 0.25; 95% CI: 0.18, 0.34, P = 0.0001], Free service charge [OR, 0.70; 95% CI: 0.57, 0.86, P < 0.0007], Privacy during examination [OR, 0.25; 95% CI: 0.10, 0.64, P < 0.004], Respectful maternal care [OR,0.40;95%CI:0.19,0.83,P=0.01], Plan to delivered at health facility [OR, 0.49; 95% CI: 0.37, 0.66, P < 0.00001] and ANC follow-up [OR, 0.39; 95% CI: 0.24, 0.63, P < 0.0001].<br>Conclusion: This review identified factors such as promptness of care, ANC follow-up, Privacy, Respectful maternal care, Plan to deliver at health facility, and fee service charge were significant predictors for client satisfaction on Labor and delivery service in Ethiopia.<br>Keywords: Client satisfaction, labor, and delivery, Ethiopia, systematic review, Meta-analysis.
Order of Authors:	REKIKU FIKRE ABEBE, BSc in midwiverey
	Kidist Eshetu
	Muntasha Berhanu
Additional Information:	
Question	Response
**Financial Disclosure**<br><br>Enter a financial disclosure statement that describes the sources of funding for the work included in this submission. Review the submission guidelines for detailed	no

Powered by Editorial Manager® and ProduXion Manager® from Aries Systems Corporation

requirements. View published research articles from *PLOS ONE* for specific examples.<br><br>This statement is required for submission and **will appear in the published article** if the submission is accepted. Please make sure it is accurate.<br><br>**Unfunded studies**<br>Enter: *The author(s) received no specific funding for this work.*<br><br>**Funded studies**<br>Enter a statement with the following details:<br>• Initials of the authors who received each award<br>• Grant numbers awarded to each author<br>• The full name of each funder<br>• URL of each funder website<br>• Did the sponsors or funders play any role in the study design, data collection and analysis, decision to publish, or preparation of the manuscript?<br>• **NO** - Include this sentence at the end of your statement: *The funders had no role in study design, data collection and analysis, decision to publish, or preparation of the manuscript.*<br>• **YES** - Specify the role(s) played.<br><br>* typeset	
**Competing Interests**<br><br>Use the instructions below to enter a competing interest statement for this submission. On behalf of all authors, disclose any competing interests that could be perceived to bias this work—acknowledging all financial support and any other relevant financial or non-financial competing interests.<br><br>This statement **will appear in the published article** if the submission is accepted. Please make sure it is accurate. View published research articles from *PLOS ONE* for specific examples.	NO

**NO authors have competing interests**<br><br>Enter: *The authors have declared that no competing interests exist.*<br><br>**Authors with competing interests**<br><br>Enter competing interest details beginning with this statement:<br><br>*I have read the journal's policy and the authors of this manuscript have the following competing interests: [insert competing interests here]*<br><br>* typeset	
**Ethics Statement**<br><br>Enter an ethics statement for this submission. This statement is required if the study involved:<br><br>• Human participants<br>• Human specimens or tissue<br>• Vertebrate animals or cephalopods<br>• Vertebrate embryos or tissues<br>• Field research<br><br>Write "N/A" if the submission does not require an ethics statement.<br><br>General guidance is provided below. Consult the submission guidelines for detailed instructions. **Make sure that all information entered here is included in the Methods section of the manuscript.**	NA

**Format for specific study types**<br><br>**Human Subject Research (involving human participants and/or tissue)**<br>• Give the name of the institutional review board or ethics committee that approved the study<br>• Include the approval number and/or a statement indicating approval of this research<br>• Indicate the form of consent obtained (written/oral) or the reason that consent was not obtained (e.g. the data were analyzed anonymously)<br><br>**Animal Research (involving vertebrate animals, embryos or tissues)**<br>• Provide the name of the Institutional Animal Care and Use Committee (IACUC) or other relevant ethics board that reviewed the study protocol, and indicate whether they approved this research or granted a formal waiver of ethical approval<br>• Include an approval number if one was obtained<br>• If the study involved *non-human primates*, add *additional details* about animal welfare and steps taken to ameliorate suffering<br>• If anesthesia, euthanasia, or any kind of animal sacrifice is part of the study, include briefly which substances and/or methods were applied<br><br>**Field Research**<br><br>Include the following details if this study involves the collection of plant, animal, or other materials from a natural setting:<br>• Field permit number<br>• Name of the institution or relevant body that granted permission	
**Data Availability**<br><br>Authors are required to make all data underlying the findings described fully available, without restriction, and from the time of publication. PLOS allows rare exceptions to address legal and ethical concerns. See the PLOS Data Policy and FAQ for detailed information.	Yes - all data are fully available without restriction

Powered by Editorial Manager® and ProduXion Manager® from Aries Systems Corporation

A Data Availability Statement describing where the data can be found is required at submission. Your answers to this question constitute the Data Availability Statement and **will be published in the article**, if accepted.<br><br>**Important:** Stating 'data available on request from the author' is not sufficient. If your data are only available upon request, select 'No' for the first question and explain your exceptional situation in the text box.<br><br>Do the authors confirm that all data underlying the findings described in their manuscript are fully available without restriction?	
**Describe where the data may be found in full sentences. If you are copying our sample text, replace any instances of XXX with the appropriate details.**<br><br>• If the data are **held or will be held in a public repository**, include URLs, accession numbers or DOIs. If this information will only be available after acceptance, indicate this by ticking the box below. For example: *All XXX files are available from the XXX database (accession number(s) XXX, XXX.*).<br>• If the data are all contained **within the manuscript and/or Supporting Information files**, enter the following: *All relevant data are within the manuscript and its Supporting Information files.*<br>• If neither of these applies but you are able to provide **details of access elsewhere**, with or without limitations, please do so. For example:<br><br>*Data cannot be shared publicly because of [XXX]. Data are available from the XXX Institutional Data Access / Ethics Committee (contact via XXX) for researchers who meet the criteria for access to confidential data.*<br><br>*The data underlying the results presented in the study are available from (include the name of the third party*	xx

and contact information or URL).<br>• This text is appropriate if the data are owned by a third party and authors do not have permission to share the data.<br><br>* typeset	
Additional data availability information:	Tick here if the URLs/accession numbers/DOIs will be available only after acceptance of the manuscript for publication so that we can ensure their inclusion before publication.

Manuscript Click here to access/download;Manuscript;manuscrpit.docx

What determines client satisfaction on labor and delivery service in Ethiopia? systematic review and meta-analysis

Rekiku Fikre Abebe[1]*, Kidist Eshetu[1], Muntasha Berhanu[2]

Authors' Affiliations

[1]Hawassa University, College of Medicine and Health Science, Department of Midwifery, P.O. Box 1560, Hawassa, Ethiopia

[1]Hawassa Health science college, Department of HIT P.O. Box 1111, Hawassa, Ethiopia.

2 Hawassa Adare General Hospital, Hospital Manager, P.o.box,2312, Hawassa, Ethiopia.

Authors email address

1. Rekiku Fikre-frekiku@yahoo.com
2. Kidist Eshetu- kideshe@gmail.com
3. Muntasha Brehanu- muntasha205@gmail.com

***Corresponding author:**

Specialty, Maternal, and, Reproductive Health Nursing, Hawassa University College of Medicine and Health Science, Department of Midwifery.

Email: frekiku@yahoo.com, (RF) Phone: +25191154598

Abstract

Introduction: The uptake of service utilization increments dictated by the satisfaction of clients and the outcome of mother and newborn. Client satisfaction is one of the vital pointers to measure the quality of servce based up on satisfaction of the client. However, it is underwhelmed and little evidence is existed in Ethiopia .Therefore, the purpose of this systematic review aimed to assess the prevalence and determinant of client satisfaction on labor and delivery service in Ethiopia.

Methodology: This studies were included published and unpublished articles. The main databases PubMed, Embase, EBSCO, Medline, CINHAL, Poplin, and the search engine like google and google scholar were used. Observational study design studies were conducted in the English language and met the eligibility criteria were included in the review. Meta-analyses with random effects were performed. Data synthesis and statistical analysis were conducted using OpenMeta and CMA version 2 software.

Results: The pooled prevalence of client satisfaction on labor and delivery service in Ethiopia was73.5% [95%CI [64.9%,82.1%]. The pooled odds ratio showed that a positive association between client satisfaction on labour and delivery service with Promptness of care [OR, 0.25; 95% CI: 0.18, 0.34, P = 0.0001], Free service charge [OR, 0.70; 95% CI: 0.57, 0.86, P < 0.0007], Privacy during examination [OR, 0.25; 95% CI: 0.10, 0.64, P < 0.004], Respectful maternal care [OR,0.40;95%CI:0.19,0.83,P=0.01], Plan to delivered at health facility [OR, 0.49; 95% CI: 0.37, 0.66, P < 0.00001] and ANC follow-up [OR, 0.39; 95% CI: 0.24, 0.63, P < 0.0001].

Conclusion: This review identified factors such as promptness of care, ANC follow-up, Privacy, Respectful maternal care, Plan to deliver at health facility, and fee service charge were significant predictors for client satisfaction on Labor and delivery service in Ethiopia.

Keywords: Client satisfaction, labor, and delivery, Ethiopia, systematic review, Meta-analysis.

Background

Perhaps the concept of quality of care is operationalized in to different dimention based on the outcome of interest, recently the focus of the health care system is shifted in to the pillar of the quality of the service based on the key performance indicators giving much emphasis on client satisfaction. Client satisfaction is one of the vital pointers to measure the quality of service based up on satisfaction of the client on the service they received, howeevr, it is measured based on the value and feedback of the client and family members based on the perceived care they received. [1-3]. Despite the advancement of technology, the expansion of health care facilities, and policy improvement to alleviate preventable causes of maternal and neonatal mortality, maternal and neonatal mortality is still high in developing countries [4].

Globally widespread attention is given on raising the coverage of life-saving intervention for mothers and babies to meet SDGs by 2030 and the Ethiopian government has made several efforts over the past decades to decrease maternal and neonatal mortality; though the efforts weren't attained the expected result as evidence the MDG targeted of MMR of 267 per 100,000 births vs 420 per 100,000 births [5-7].

In developing countries, including Ethiopia several strategies were exercised: like increasing the coverage of skilled birth attendant and the expansion facility-based maternity care as a means of reduction of maternal mortality, Yet the quality of care is still poor due to various reasons like lack of equipment, shortage of manpower and gap of providers treating clients with compationate and respectful care [8].

There is substantial improvement in access to essential health services, nevertheless, emotional aspect of clients is ignored and leads them to poor satisfaction and low facility utilization attributed to high levels of maternal and child mortality [9,10].

Several studies show that most women had faced so many problems during labor and delivery including insulting and lack of Compationate and respectful care by care providers. In such a way by one or another, the right of the women was diminished and the practice of the providers become unethical [11-16].

Event though, perceived satisfaction of clients depends on the outcome of the mother and neonate however, this alone didn't determine the quality of service [17,18]. The perceived satisfaction of clients has a positive impact to increase service uptake, pararaley dissatisfied clients less prone to visit health care facilities so that it contributes to maternal mortality [19].

Satisfaction with maternal care service is important both to the mother, infant's and for the family which is evidenced by different Studies showing mother's positive perception of birth experience has been linked to positive feelings toward her infant and adaptation to the mothering role [21-22]. There are evidences shows that determinants of client satisfaction on labor and delivery service in developed nations, whereas there is lack of pooled evidence in Ethiopia [23-26]. Therefore, this study aimed to summarize the evidence of determinants of client satisfaction on labor and delivery service in Ethiopia. The summarized evidence obtained from this study helps the concerned bodies to identify existing gaps and propose strategies to increase client satisfaction in Ethiopia.

Methods

Study protocol

The protocol for this systematic review and meta-analysis has been registered in the International Prospective Register of systematic reviews (PROSPERO). The methodology of this systematic review and meta-analysis was once developed via following the Preferred Reporting Items for Systematic Reviews and Meta-Analyses (PRISMA)**.**

Inclusion criteria

Study design and period

Observational studies reporting client/women/mother satisfaction on Labor and delivery service in Ethiopia published After 2010 and before Dec 2019 were considered. Only English language full-text reports were included.

Study setting

Institutional /community-based studies considering satisfaction.

Participants: Postnatal mother/women

Exposure: Predictors/determinants/factors of Client satisfaction.

Outcome: client/women/mother satisfaction on labor and delivery service

Eligibility criteria

The study participants has been postnatal mothers/women in Ethiopia. The investigators encomposs observational studies that have been conducted at a facility/community setting in different parts of Ethiopia on the client/women/mother satisfaction on labor and delivery service. Besides, studies that were published after 2010 and were avaliable till Dec 2019 and written in English have been eligible for this systematic review and meta-analysis.

Articles with irretrievable full texts, records with unrelated outcome measures and articles with missing or insufficient outcomes had been excluded. Reviews, commentaries, editorial, case series/reports, and patient stories had been excluded from the systematic review.

Sources of studies and searching strategies

The authors performed systematic literature searches from the authentic major electronic databases such as MEDLINE, PubMed, EMBASE, Emcare, CINAHL (EBSCOhost), Web of Science, Scopus, Poplin and diffrent literature sources including Google Scholar. Also, the hand (manual) search of various repositories was performed to retrieve unpublished studies and gray literature. We used MeSH terms, key terms and search strings by extracting from the review questions for all the searches.

Advanced search strategies were applied in major databases to retrieve relevant findings closely related to client/women/mother satisfaction on labor and delivery service in Ethiopia. The search was conducted with the aid of carefully selected keywords and indexing terms.

The search strategy included “Factors” OR “Associate” OR "related factors “OR “determinants” OR “Predictors” AND "client satisfaction “OR “women satisfaction” OR "mother satisfaction’’ “AND "labor “AND ‘’ delivery’’ AND “Ethiopia". Both authors constructed the search strings (RF and MB). The overall search result was compiled using EndNote X9 citation manager software **Additional file 1**.

Selection of studies

All search results were exported to the EndNote X9 citation manager and duplicated studies were removed. Later, articles were screened through a careful reading of the title and abstract. The two authors screened and evaluated the studies independently.

The titles and abstracts of studies that mentioned the outcomes of the review were considered for further evaluation to be included in the systematic review and meta-analysis. Then the full-texts of the retained studies were further evaluated based on the quality of their objective, methods, participants/population, and key findings.

The authors (RF, KE, and MB) independently evaluated the quality of the studies included against the Joanna Briggs Institute (JBI) critical appraisal tool for cross-sectional studies checklists. In case of disagreement between the quality assessment results of the two authors, the differences were resolved by consensus for inclusion. The overall study selection process is presented using the PRISMA statement flow diagram (**fig 1**).

Data extraction and recording

Findings from the selected studies were extracted and stored using data extraction template prepared on Microsoft Word and then to Excel (2016), followed by extraction of important data based on study characteristics (Region, first author, year of publication, study design, and outcome of interest) by the two authors independently. For the prevalence of client satisfaction, we used unconverted proportional data to calculate the proportion/prevalence in percentage using OpenMeta software. Most of the determinants of the client satisfaction summary measures were done by the pooled odds ratio using Comprehensive meta-analysis (CMA) software.

Risk of bias assessment

The methodological reputability and quality of the findings of the included studies were critically evaluated using the quality assessment tool for observational studies cross-sectional developed by the Joanna Briggs Institute (JBI). To ensure quality, the investigators searched for studies using a comprehensive strategy (electronic databases, and manual search) which included published and/or unpublished studies.

To minimize bias, the two authors independently screened the studies using clear objective eligibility criteria. Publication bias-was explored using visual inspection of the funnel plot. Additionally, Egger's regression test was carried out to check the statistical symmetry of the funnel plot**.**

Critical appraisal of studies

The methodological reputability and quality of the findings of the included studies were critically evaluated using the quality assessment tool for observational studies developed by the Joanna Briggs Institute (JBI). The two authors (RF and KE) independently evaluated the quality of the studies. The mean score of the two authors was taken for a final decision. The differences in the inclusion of the studies were resolved by consensus. The cross-sectional observational studies checklist was graded out of 9 points. The included studies were evaluated against each indicator of the tool and categorized as high-, moderate-, and low quality. A high-quality score above 80%, moderate-quality between 60%-80%, and low-quality below 60%. Studies with a score greater than or equal to 60% were included. This critical appraisal was conducted to assess the internal validity (systematic error) and external validity (generalizability) of studies and to reduce the risk of biases.

Strategy for data synthesis

The findings of the included studies were first presented using a narrative synthesis. Study descriptions and summary (author-year, region, aim, design and population, sample size, and key findings) were compiled using Microsoft word. The raw numerical data prevalence (n) and total sample size (N)) from each study were extracted and recorded on a Microsoft word then exported to an Excel Spreadsheet. The authors conducted data synthesis and statistical analysis.

Meta-analysis was conducted using OpenMeta and CMA version 2 software to compute the pooled prevalence and determinates with client satisfaction on labor and delivery service.

A meta-analysis of observational studies was conducted, based on recommendations made by Higgins et al. (An I^2 of 75/100%, suggesting considerable heterogeneity). In the meantime, heterogeneity between the included studies was examined using the I^2 statistic.

Therefore, the presence of heterogeneity between studies was assumed if the I^2 statistic greater than seventy-five percent. A random-effects model was used to determine the pooled prevalence and determinates with client satisfaction on labor and delivery service in Ethiopia.

RESULT

Review Studies

over-all of 87 articles have been identified through the main electronic databases and additional applicable sources search from January 1/2020 to Febraruary1/2/2020. From all identified studies, 29 articles were removed due to duplication whilelst 58 studies have been reserved for further screening. Of these, 24 have been excluded after being screened according to titles and abstracts. Of the 34 remaining articles, 18 studies had been excluded due to inconsistency with the inclusion criteria set for the review. Finally, 16 studies that fulfilled the eligibility criteria were included for the systematic review and meta-analysis. General characteristics and descriptions of the studies chosen for the meta-analysis have been outlined in **(Table 1*).***

Prevalence of Client satisfaction on labor and delivery service

The pooled approximation of the magnitude of client satisfaction in Ethiopia was 73.5% [95%CI [64.9%,82.1%] (Fi***g.2***).

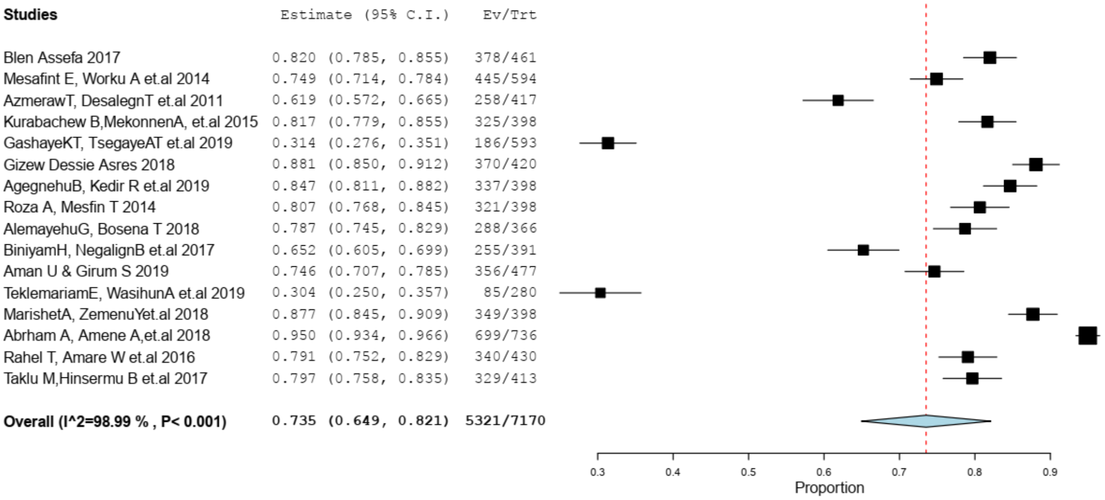

Figure 2*:* Pooled prevalence of client satisfaction on labor and delivery service in Ethiopia, 2020.

Determinants of client satisfaction on labour and delivery service

The results of this review have shown determinants significantly associated with client satisfaction on labour and delivery service in Ethiopia, Disrespectful maternal care [OR,0.40;95%CI:0.19,0.83,P=0.01] waiting time more than one hour [OR, 0.25; 95% CI: 0.18, 0.34, P = 0.0001], Service charge [OR, 0.70; 95% CI: 0.57, 0.86, P < 0.0007], lack of privacy during examination [OR, 0.25; 95% CI: 0.10, 0.64, P < 0.004], Not any plan to delivered at health facility [OR, 0.49; 95% CI: 0.37, 0.66, P < 0.00001]and No ANC follow-up [OR, 0.39; 95% CI: 0.24, 0.63, P < 0.0001] were less likely associated with client satisfaction on Labor and delivery service in Ethiopia.

Respectful maternal care

The findings of the review indicated a significant association between respectful maternal care and client satisfaction. Disrespectful maternal care were 0.40 times less likely satisfied compared to respectful maternal care [OR=0.40;95% CI: 0.19, 0.83, P = 0.01]. Heterogeneity test indicated I2 = 75%, (**Fig3**)

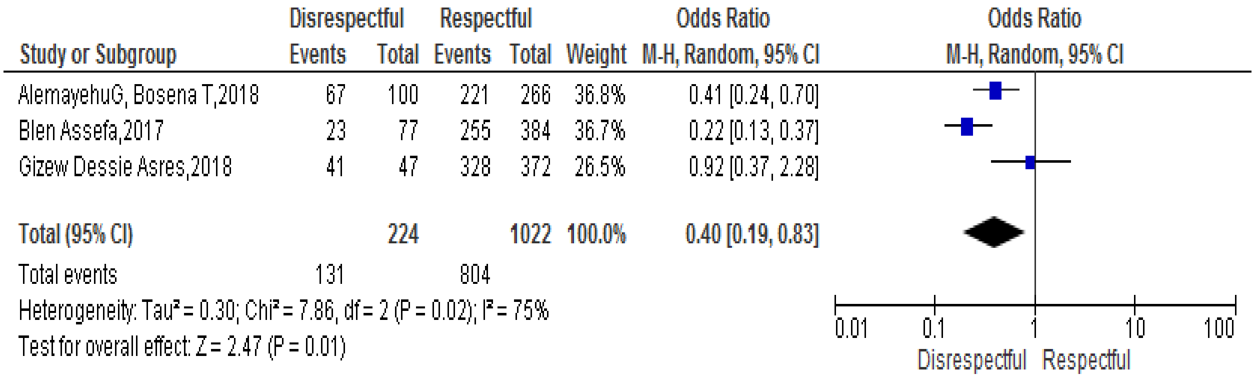

Fig3: Assoscation bettween respectful maternal care with client satisfaction in Ethiopia ,2020.

Promptness of care

The findings of the review indicated a significant association between delay to receive care and client satisfaction. Clients who wait more than one hour to receive care were 0.25 times less likely satisfied compared to clients who received care with less than an hour [OR=0.25;95% CI: 0.18, 0.34, P = 0.0001]. Heterogeneity test indicated I2 = 0%, ***(*****Fig4*****)***.

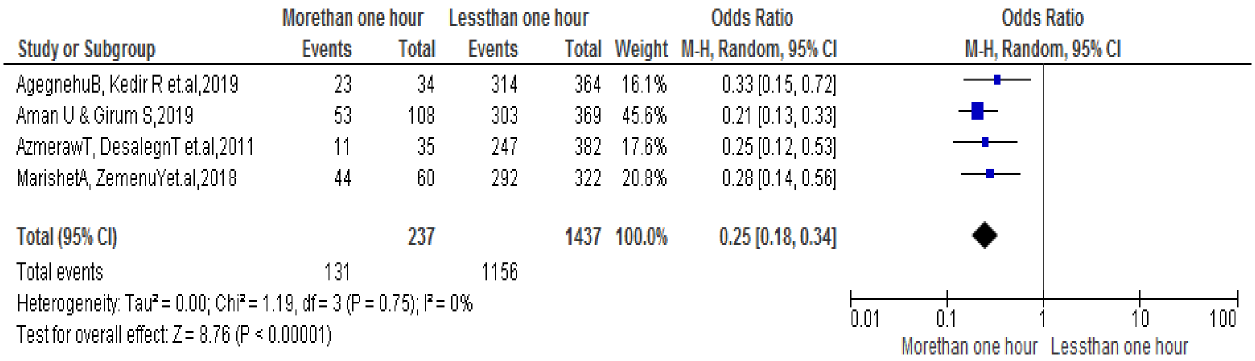

Fig4: Association between delay to receive care with client satisfaction in Ethiopia,2020.

Service fee

This review demonstrated that there was significant association between Service charge and client satisfaction in the random model [OR, 0.70; 95% CI: 0.57, 0.86; P = 0.0007]. clients who were charged for the service were 0.70 times less likely satisfied as compared to clients who had received care free of charge (**Fig *5*).**

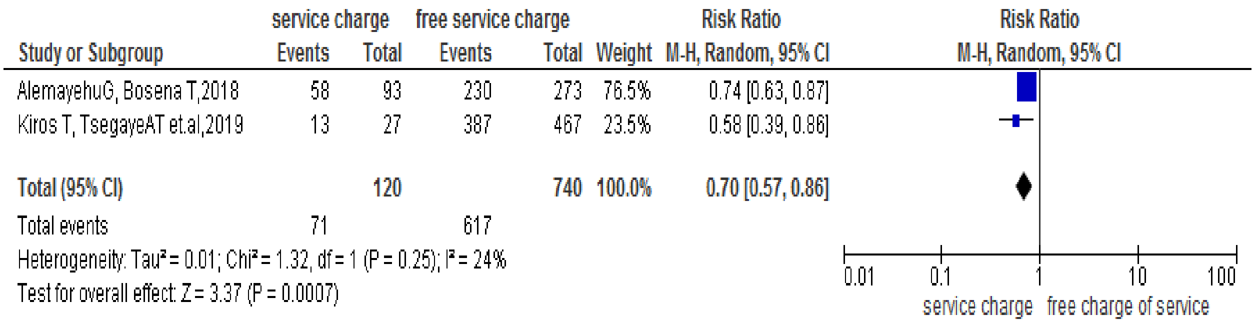

Fig5:Association betweeen service charge with client satisifaction in Ethiopia,2020.

Privacy

Privacy maintained during the examination was significantly associated with client satisfaction, the odds of client satisfaction were high among those who maintained privacy as compared to others [OR, 0.25; 95% CI: 0.10, 0.64, P=0.004]. Clients who lack privacy were 0.25 times less likely satisfied as compared to those who maintained privacy. But considerable heterogeneity was found too high (I^2 = 92%), hence the random effect model was assumed in the analysis. Sensitivity was done of analysis but did not bring significant change in the overall summary results of OR **(Fig 6*).***

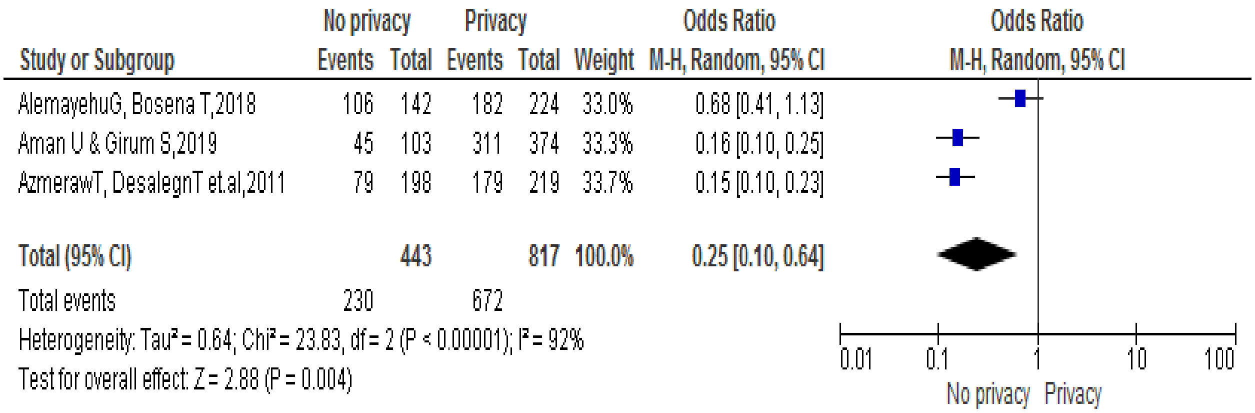

Fig6: Association between privacy with client satisfaction in Ethiopia,2020.

ANC follow-up

The findings of the review indicated a significant association between ANC follow-up and client satisfaction. Clients with No ANC follow-up were 0.39 times less likely satisfied compared to ANC follow-up [OR=0.39;95% CI: 0.24, 0.63, P = 0.0001]. Heterogeneity test indicated I2 = o%, (**Fig7**)

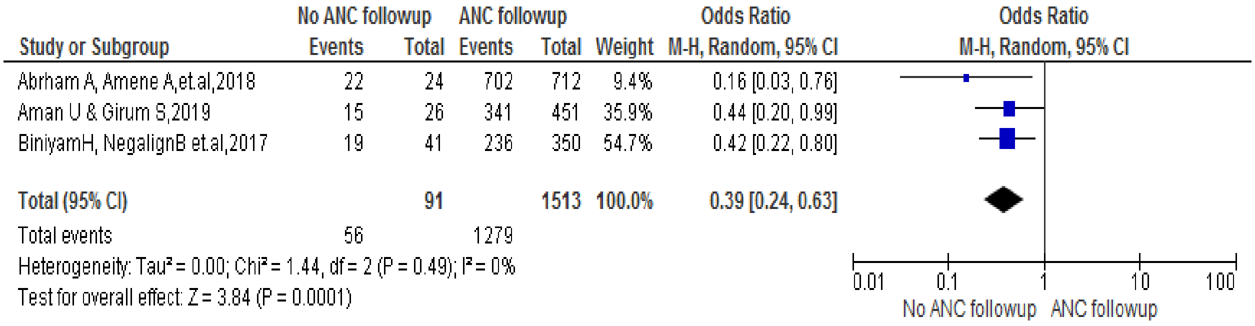

Fig7: Association between ANC follow-up with client satisfaction in Ethiopia,2020.

Plan to deliver at the facility

The results of the review presented there was statistically significant association between clients who had the plan to deliver at the health facility and client satisfaction [OR, 0.49; 95% CI: 0.37,

66, P<0.00001]. Clients who had no plan to deliver at the health facilities were 0.49 times less likely satisfied than their counter (Fig8).

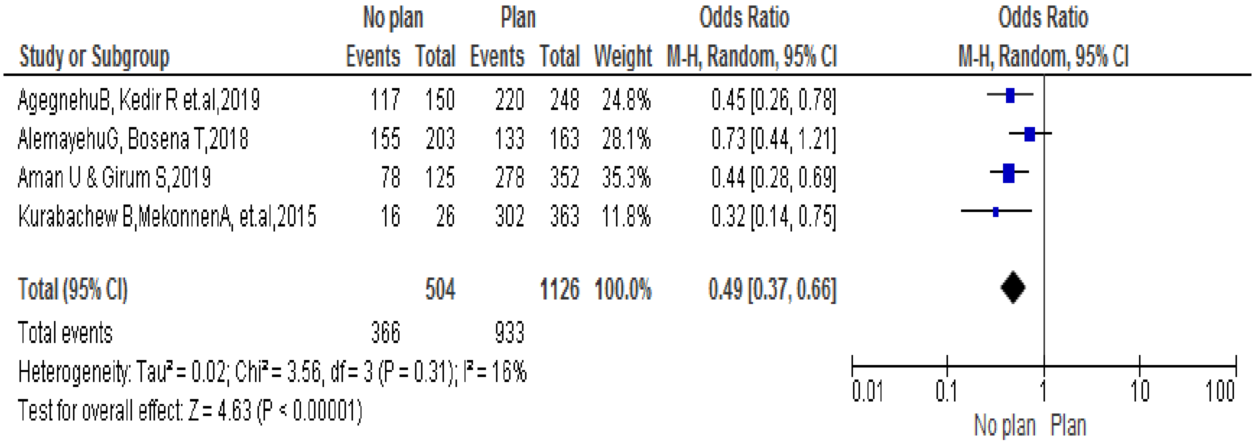

Fig 8: Association between Plan to deliver at a health facility with client satisfaction in Ethiopia,2020.

Publication bias

To check publication bias among the included studies for the meta-analysis, funnel plot and Egger's test were carried out. (**Fig** 9&10)

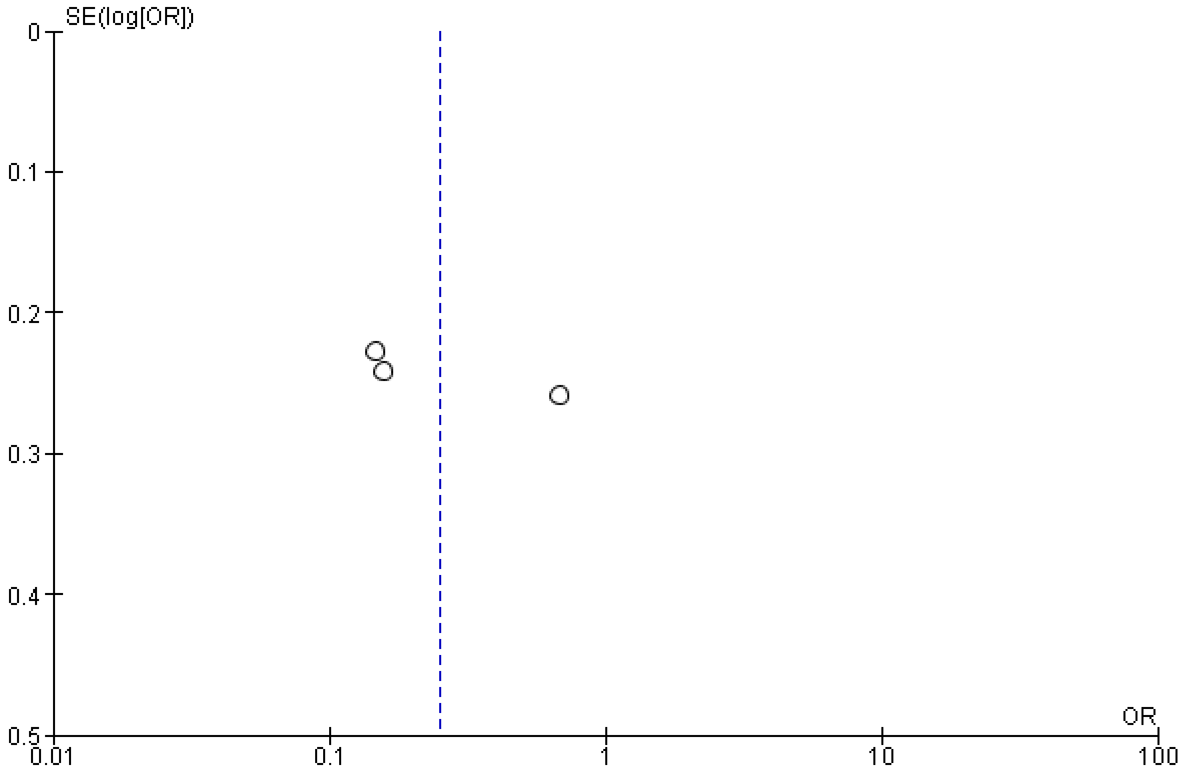

Fig 9. Publication bias on the privacy of the clients during an examination.

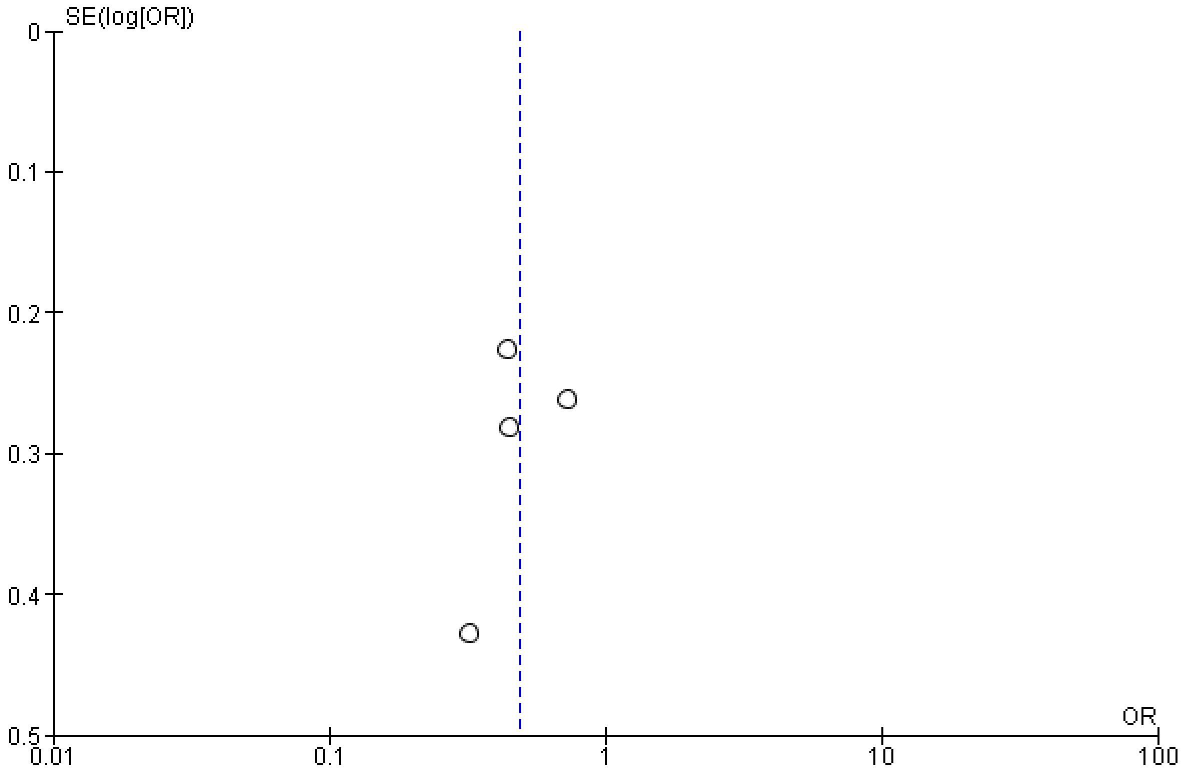

Fig10. Publication bias on a plan for delivery at a health facility.

Discussion

Evaluating client satisfaction remains one of the key performance indicators to control the quality of services within a facility which provides maternal care. This comprehensive study provides potted information on determinants that affect client satisfaction on labor and delivery service in Ethiopia. This review incorporated studies with methodological quality and all of the studies were descriptive cross-sectional.

This systematic and meta-analysis revealed that the overall prevalence of client satisfaction on labor and delivery service during the period studied in Ethiopia was 73.5% (95 CI: 64.9 82.1). This result is highest than studies in Eritrea 20.8% [27] and Kenya 54.5% [28]. The possible reason for this might be due to policy difference that all maternal service was free of charge, competency of providers and the expansion of training corresponding EMONC, companionate and respectful care for MCH providers. However, client satisfaction in our meta-analysis was lower than the study done in Nepal 86% [29] and Ghana 80% [30]. The difference might be due to poor job satisfaction of providers, due to an inadequate number of providers.

Our analysis revealed that the magnitude of client satisfaction across the region shows few discrepancies in Oromia 74.8%, SNNPR 73.1% and in Amhara 67.6%. The possible reason for this discrepancy due to the difference in the standard of care, the difference in exercising compassionate and respectful maternal care and might be due to the presence of experienced providers. In this review and meta-analysis, we found several determinants that had a significant association with client satisfaction on labor and delivery service in Ethiopia. The current review indicated that National policy, structural and process play a significant role in client satisfaction on labor and delivery service. This meta-analysis shows that client satisfaction on labor and

delivery service was positively associated with, respectfulness of providers, promptness of care, privacy, plan to deliver at the facility, free service charge, and ANC follow-up.

This review revealed that the client who received respectful maternal care by providers was positively associated with client satisfaction on labor and delivery. [31-33]. A similar result was seen from another primary study in Zambia, Malawi, Vietnam, Oman, and southeast Nigeria and systematic review in developing countries, where client satisfaction on labor and delivery were positively associated with respectfulness of providers. [34-40]. This is due to, clients treated based on the principle of women-centered approaches, by curiosity, empathy so that it can allow exercising their right and to receive a high level of quality of services. In contrast a study in Pakistan, Turkey and Ghana it was revealed that the absence of a respectful approach by providers increased the odds of dissatisfaction of clients on labor and delivery service [41-43].

In this review privacy during examination increased client satisfaction. [33,44,45]. A systematic review in developing country it was reported that privacy during examination increases client satisfaction on labor and delivery service [39]. The possible reason for this due to associated with it respect the cultural norm of the people avoids shame and it builds confidence. In a study in n Eritrea, Kenya, Nepal, Bangladesh, and India revealed a lack of privacy increased the odds of dissatisfaction of clients on labor and delivery service [27-29, 46,47].

Besides, free service charge was among the determinants contributing to client satisfaction on labor and delivery service in Ethiopia [33,49]. This review found was a similar study conducted in Kenya, Nepal, and Gambia [28,29,48]. This might be due to most clients were poor socioeconomic status, so it facilitates room to use the money for other needs that may arise during their pregnancy-child birth process. In contrast a study in India it was revealed that service charge for labor and delivery service was positively associated with client satisfaction [47].

The review revealed that promptness of care was associated with client satisfaction. [44,45,49,51]. This was in line with a study in Oman, Nigeria, Bangladesh and India and systematic review in developing countries [36,37,39,52]. The possible reason might be it is the time when the client needs special attention and time makes to receive the quality of standard service.

In this review ANC follow-up was associated with client satisfaction [45,53,54]. The possible reason might be due to care received by the same providers who followed during ANC and similar environment so it makes it women-friendly.

The plan to delivered at the health facility was positively associated with client satisfaction [33,45,49,55]. The possible reason for this might be based on free transportation/ambulance access, compassionate and respectful care received during the antepartum period and pre-planned scheduled for every expected outcome.

Conclusion

This review identified determinates for client satisfaction on Labor and delivery service in Ethiopia. Based on our review waiting time, cost of the service, Respectfulness of the providers, Privacy, plan to deliver at the health facility and ANC follow-up all increased client satisfaction on Labor and delivery service. This meta-analysis has certain limitation, all of the studies included were cross-section so that it restricts us to do cause-effect analysis. The included study has different in the quality of study which is observed by heterogeneity.

Declaration

Abbreviation

CMA------------------------ Comprehensive meta analysis

PRISMA-------------------- Preferred Reporting Items for Systematic Reviews and Meta-Analyses

JBI---------------------------- Joanna Briggs Institute

Ethical approval and consent

Not-applicable

Consent for publication

Not-applicable

Availability of data and materials

If we request, we can avail all the data we used.

Competing interests

The authors declared that have no competing interests.

Funding

Not-available

Authors'contributions

RF, KE, and MB conceived and designed the review. RF, KE and MB carried out the draft of the manuscript and KE is the guarantor of the review. RF, KE and MB developed the search strings. RF, KE and MB screened and selected studies. RF and KE extracted the data. RF and MB evaluated the quality of the studies', RF and MB carried out analysis and interpretation. RF, KE, and MB rigorously reviewed the manuscript. All authors read and approved the final version of the manuscript.

Acknowledgments

We would like to thank the College of medicine and health science, Hawassa University (Ethiopia) for non-financial support.

REFERENCE

1. Donabedian A: An Introduction to Quality Assurance in Health Care. Oxford University Press, USA; 2002.
2. Donabedian A: The quality of care. How can it be assessed? 1988. Archives of Pathology & Laboratory Medicine 1997, 121(11):1145-1150.
3. West away MS RP, Vanzyl D, Seager JR: Interpersonal and organizational dimensions of patient satisfaction. International journal for quality in health care 2003, 15(4):337-344.
4. World health statistics 2016: monitoring health for the Sustainable Development Goals. Geneva: World Health Organization; 2016 (http://who. int/gho/publications/world_health_ statistics/2016/EN_WHS2016_TOC.pdf, accessed 24 February 2018).
5. World health Organization. Strategies toward ending preventable maternal mortality (EPMM), vol. 6736. Geneva: WHO; 2015. p. 1–4.
6. World Health Organization. Health in 2015: from MDGs, Millennium Development Goals to SDGs, Sustainable Development Goals, vol. 204. World Health Organization; 2015.
7. EDHS (2016). "Ethiopia Demographic and Health Survey 2016." Addis Ababa, Ethiopia,2016.
8. Jahn A, Dar Iang M, Shah U, Diesfeld HJ. Maternity care in rural Nepal: a health service analysis. Tropical Med Int Health. 2000;5(9):657–65
9. Austin A, Langer A, Salam RA, Lassi ZS, Das JK, Bhutta ZA: Approaches to improve the quality of maternal and newborn health care: an overview of the evidence. Reprod Health 2014, 11(Suppl 2):S1.
10. World Health Organization. Delivering quality health services. Geneva: 2018. Available from: http://apps.who.int/bookorders.

11. World Health Organization (2005) RESPECTFUL MATERNITY CARE: THE UNIVERSAL RIGHTS OF CHILDBEARING WOMEN. Washington DC: White Riban Alliance.
12. WHO (2015) The prevention and elimination of disrespect and abuse during facility-based childbirth. Geneva, Switzerland: human production program.
13. Bowser D, Hill K (2010) Exploring Evidence for Disrespect and Abuse in Facility-Based Childbirth. USAID.
14. Bohren MA, Vogel JP, Hunter EC, Lutsiv O, Makh SK, et al. (2015) The Mistreatment of Women during Childbirth in Health Facilities Globally: A Mixed-Methods Systematic Review. PLoS Med 12: e1001847. pmid:26126110
15. Dzomeku MV (2011) Maternal satisfaction with care during labour: A case study of the Mampong-Ashanti district hospital maternity unit in Ghana. International Journal of Nursing and Midwifery 3: 30–34.
16. Rudman A (2007) Women's evaluation of intrapartum and postpartum care. Stockholm, Sweden: Karolinska institute. 1–76
17. Haddad S, Fournier P, Machouf N, Yatara F. What does quality mean to lay people? Community perceptions of primary care services in Guinea. Soc Sci Med. 1998;47:381–94. doi: 10.1016/S0277-9536(98)00075-6. [PubMed] [CrossRef] [Google Scholar]
18. Andaleeb SS. Service quality perceptions and patient satisfaction: a study of hospitals in a developing country. Soc Sci Med. 2001; 52:1359–70. doi: 10.1016/S0277-9536(00)00235-5. [PubMed] [CrossRef] [Google Scholar]
19. Mpembeni RNM, Killewo JZ, Leshabari MT, Massawe SN, Jahn A, Mushi D, et al. Use pattern of maternal health services and determinants of skilled care during delivery in Southern Tanzania: implications for achievement of MDG-5 targets. BMC Pregnancy

Childbirth. 2007;7:29. doi: 10.1186/1471-2393-7-29. [PMC free article] [PubMed] [CrossRef] [Google Scholar]

20. MacKeith N, Chinganya OJM, Ahmed Y, Murray SF: Zambian women's experiences of urban maternity care: results from a community survey in Lusaka. African Journal of Reproductive Health 2003, 7(1):92-102.
21. Goodman P, Mackey MC, Tavakoli AS: Factors related to childbirth satisfaction. Journal of Advanced Nursing 2004, 46(2):212-219.
22. Simkin P: Just another day in a woman's life? Part II: Nature and consistency of women's long-term memories of their first birth experiences. Birth (Berkeley, Calif) 1992, 19(2):64-81.
23. Redshaw M, Heikkila K (2010) Delivered with care: a national survey of women's experience of maternity care USA: The National Perinatal Epidemiology Unit, University of Oxford, Old Road Campus, Oxford OX3 7LF. 61 p.
24. Tayelgn A, Zegeye DT, Kebede Y (2011) Mothers' satisfaction with referral hospital delivery service in Amhara Region, Ethiopia. BMC pregnancy & childbirth 11: 1–7.
25. Rudman A (2007) Wemen's evaluation of intrapartum and postpartum care.
26. Yohannes B, Tarekegn M, Paulos W (2013.) Mothers' Utilization Of Antenatal Care And Their Satisfaction With Delivery Services In Selected Public Health Facilities Of Wolaita Zone, Southern Ethiopia. INTERNATIONAL JOURNAL OF SCIENTIFIC & TECHNOLOGY RESEARCH 2: 74–85
27. Meron Mehari Kifle,Filmon Abraham Ghirmai, Soliana Amanuel Berhe, Winta Sium Tesfay,Yodit Teklemariam Weldegebriel,and Zebib Tesfamariam Gebrehiwet, Predictors of Women's Satisfaction with Hospital-Based Intrapartum Care in Asmara Public Hospitals, Eritrea, Hindawi Obstetrics and Gynecology International Volume 2017.

28. C. M. Gitobu , P. B. Gichangi, and W. O. Mwanda, Satisfaction with Delivery Services Offered under the Free Maternal Healthcare Policy in Kenyan Public Health Facilities, Hindawi Journal of Environmental and Public Health Volume 2018.

29. Yuba Raj Paudel, Suresh Mehata, Deepak Paudel, Maureen Dariang, Krishna Kumar Aryal, Pradeep Poudel,1 Stuart King, and Sarah Barnett, Women's Satisfaction of Maternity Care in Nepal and Its Correlation with Intended Future Utilization, Hindawi Publishing Corporation International Journal of Reproductive Medicine Volume 2015.

30. Ndeogo Amadu abdallah, factors influencing women's satisfaction with child birth services in the tamale metropolis, university of Ghana http://ugspace.ug.edu.gh2018.

31. Blen Assefa, Maternal satisfaction with delivery services of public health centers in Addis Ababa, Ethiopia, 2017.

32. Gizew Dessie Asres, Satisfaction of maternal care among women delivered at Asrade Zewude Memorial PrimaryHospital,Bure, West Gojjam, Amhara, Ethiopia: J. Public Health Epidemiol., Vol. 10(5), pp. 147-154, May 2018.

33. Alemayehu Gonie*, Bosena Tebeje, and Makeda Sinaga, Satisfaction towards Skilled Delivery Services and Associated Factors among Mothers who Gave Birth at Government Health Facilities, Jimma Town, Ethiopia, Clinics Mother Child Health 2018, 15:3.

34. MacKeith N, Chinganya OJM, Ahmed Y, Murray SF. Zambian women's experiences of urban maternity care: results from a community survey in Lusaka. Afr J Reprod Health. 2003; 7:92–102.

35. Changole J, Bandawe C, Makanani B, Nkanaunena K, Taulo F, Malunga E, et al. Patients' satisfaction with reproductive health services at Gogo Chatinkha Maternity Unit, Queen Elizabeth Central Hospital, Blantyre, Malawi. Malawi Med J. 2010; 22:5–9.

36. Duong DV, Binns CW, Lee AH, Hipgrave DB. Measuring client-perceived quality of maternity services in rural Vietnam. Int J Qual Health Care. 2004; 6:447–52.
37. Ghobashi M, Khandekar R. Satisfaction among expectant mothers with antenatal care services in the musandam region of Oman. Sultan Qaboos Univ Med J. 2008; 8:325–32.
38. Uzochukwu BSC, Onwujekwe OE, Akpala CO. Community satisfaction with the quality of MCH services in Southeast Nigeria. East Afr Med J. 2004; 81:293–9.
39. Aldana JM, Piechulek H, Al-Sabir A. Client satisfaction and quality of health care in rural Bangladesh. Bull World Health Organ. 2001; 79:512–7
40. Vera H. The client's view of high-quality care in Santiago, Chile. Stud Fam Plann. 1993; 24:40–9.
41. Kazmi S. Pakistan: consumer satisfaction and dissatisfaction with maternal and child health services. World Health Stat Q. 1995; 48:55–9.
42. Turan JM, Bulut A, Nalbant H, Ortayh N, Akalin AAK. The Quality of hospital-based antenatal care in Istanbul. Stud Fam Plann. 2006;37:49–60.
43. Avortri GS, Beke A, Nkrumah GA. Predictors of satisfaction with child birth services in public hospitals in Ghana. Int J Health Care Qual Assur. 2011;24:223–37.
44. Azmeraw Tayelgn1 Desalegn T Zegeye and Yigzaw Kebede, Mothers' satisfaction with referral hospital delivery service in Amhara Region, Ethiopia, BMC Pregnancy and Childbirth 2011, 11:78.

45. Aman Urgessa Edaso, Girum Sebsibie Teshome, Mothers' satisfaction with delivery services and associated factors at health institutions in west Arsi, Oromia regional state, Ethiopia. *MOJ Womens Health.* 2019;8(1):110–119.
46. Aldana JM, Piechulek H, Al-Sabir A. Client satisfaction and quality of health care in rural Bangladesh. Bull World Health Organ. 2001;79:512–7.
47. Das P, Basu M, Tikadar T, Biswas GC, Mirdha P, Pal R. Client satisfaction on maternal and child health services in rural Bengal. Indian J Community Med. 2010; 35:478–81.
48. Cham M, Sundby J, Vangen S. Availability and quality of emergency obstetric care in Gambia's main referral hospital: women-users' testimonies. Reprod Health. 2009;6:5
49. Agegnehu Bante getenet1,Kedir Teji Roba2,Berhanu seyoum endale3,abera Mersha Mamo1 Rasha Darghawth4, Women's satisfaction with intrapartum care and its predictors at harar hospitals, eastern Ethiopia, Nursing: Research and Reviews 2019:9 1–11.
50. Gashaye KT, Tsegaye AT, Shiferaw G, Worku AG, Abebe SM (2019) Client satisfaction with existing labor and delivery care and associated factors among mothers who gave birth in university of Gondar teaching hospital; Northwest Ethiopia: Institution based cross-sectional study. PLoS ONE 14(2): e0210693. https://doi.org/ 10.1371/journal.pone.0210693.
51. Marishet Agumasie1*, Zemenu Yohannes1 and Teferi Abegaz2, Maternal Satisfaction and Associated Factors on Delivery Care Service in Hawassa City Public Hospitals, South Ethiopia, Gynecol Obstet (Sunnyvale) 2018, 8:5.
52. Mesafint Ewunetu Mekonnen1, Worku Awoke Yalew2 and Zelalem Alamrew Anteneh2*, Women's satisfaction with child birth care in Felege Hiwot Referral Hospital, Bahir Dar city, Northwest Ethiopia, 2014, *BMC Res Notes (2015) 8:528.*

53. Biniyam Haile Tadesse, Negalign Birhanu Bayou, Gebeyehu Tsega Nebeb, Mothers' Satisfaction with Institutional Delivery Service in Public Health Facilities of Omo Nada District, Jimma Zone, Clinical Medicine Research 2017; 6(1): 23-30.
54. Abrham Abeto Temamo, Amene Abebe and Amare Admasu Menta, Mothers' Satisfaction with Institutional Delivery Service and Associated Factors among Women Attending Hospitals in Wolaita Zone Administration, SNNPR, Ethiopia, J Nutr Diet Pract, Volume 2(2): 2018.
55. Kurabachew Bitew,1 Mekonnen Ayichiluhm,2 and Kedir Yimam, Maternal Satisfaction on Delivery Service and Its Associated Factors among Mothers Who Gave Birth in Public Health Facilities of Debre Markos Town, Northwest Ethiopia, BioMed Research International Volume 2015.

Click here to access/download
Supporting Information
Quality assessment for included study.docx

Click here to access/download
Supporting Information
cheeclist-prisuma.docx

Click here to access/download
Supporting Information
addtional file.docx
