# Supplementary material for: What determines client satisfaction on labor and delivery service in Ethiopia? systematic review and meta-analysis
Source: PLoS One. 2021 Apr 22;16(4):e0249995. doi: 10.1371/journal.pone.0249995 (PMC8061977; doi:10.1371/journal.pone.0249995)
Supplement: Attachment — Submitted filename: Comments on Manuscript - 29 Sept 2020.docx [file pone.0249995.s004.docx]

**Abstract** – would like to see it formatted a bit more clearly, perhaps leaving at least one line between the sections so that it is easier to read:

- Background
- Methods
- Results
- Conclusion

*Background* – there are sentences that do not make sense, please see highlights in yellow in the manuscript

*Methods* – when were the databases searched?

*Conclusion* – is a repeat of the results, the conclusion should answer the question, “So what?”. You have obtained the results that you have obtained. So what should we know or do about this?

**Background**

*Generally*, the background section could be improved by focusing on what we know about determinants of client satisfaction on labour and delivery services, perhaps starting globally and then focusing on Ethiopia. I would have liked to see some of the following question addressed in the background section:

- What is client satisfaction and why is it important?
- How is client satisfaction measured?
- Is there an association between client satisfaction and uptake of labour and delivery services?
- What other factors are likely to affect the uptake of labour and delivery services?
- Why is this study important – why was it important to conduct a systematic review on client satisfaction of labour and delivery services in Ethiopia?
- Why a systematic review?

*Specifically*

Sentence starting with, “Despite the advancement of technology….” Seems to be misplaced here. The authors seem to be suggesting that neonatal mortality is high because of the reasons given, but none of these reasons appear to be related to client satisfaction. Is there some way that they can relate neonatal mortality to client satisfaction if the literature review points to this?

Sentence starting with, “In developing countries including Ethiopia…” you mention that several strategies were exercised. This part of the sentence seems incomplete: several strategies were exercised to do what?

Methods

Authors have done a lot of work in conducting this systematic review and meta analysis and they give a good description of the methodology in the manuscript. All the information is available, and whilst I find it interesting, somehow it also difficult to read. Might the authors want to continue a slight reorganisation of this section thus:

- Objectives of the review
- Eligibility criteria where they have the following:
  - Participants
  - Settings
  - Interventions and outcomes
  - Types of studies
  - Time period
- Information sources and search strategy
- Study Selection
- Quality Assessment of Included Studies
- Data Collection Process

And then, at the end of the section, they can write something like the following:

*The systematic review has been designed and reported according to the PRISMA 230 checklist (****ref****). The systematic review protocol has been published (****ref****) and registered in the 231 ‘International Prospective Register for Systematic Reviews’ (PROSPERO) (****ref****).*

**Results**

Plan to deliver at the facility – last sentence in this section has “counter”. Not sure what this means in this sentence.

**Discussion and Policy Implications** – I would like to see the discussion section arranged thus:

- Summary of main results
- Overall completeness and applicability of evidence
- Quality of the evidence
- Potential biases in the review process
- Agreements and disagreements with other studies or reviews
- Implications for practice
- Implications for research

**Conclusion** – sentence on limitation should be in Discussion section

Please see my comments about the Conclusion in the abstract section. This section should be telling us what the results mean and what we can do with them. Now that we have the results that we have, what can we do about it?

***Language, spelling mistakes, and readability***

- Text highlighted in yellow is difficult to understand
- Text highlighted in green are spelling mistakes or text which should not be in this format: sometimes there are capital letters in the middle of a sentence or words have been abbreviated when they should not have been
- All acronyms must be spelt out the first time they are used, and afterwards just the acronym, for example, Sustainable Development Goals (SDGs). After this, it is admissible to use just SDGs.
- No abbreviations such as “weren’t” – it should be spelt out in full
- Cannot start a sentence with “But…” in the results
