# Supplementary material for: What determines client satisfaction on labor and delivery service in Ethiopia? systematic review and meta-analysis
Source: PLoS One. 2021 Apr 22;16(4):e0249995. doi: 10.1371/journal.pone.0249995 (PMC8061977; doi:10.1371/journal.pone.0249995)
Supplement: Attachment — Submitted filename: Response to reviwers.docx [file pone.0249995.s007.docx]

**Manuscript Number: PONE-D-20-11487R1**

Response to the Academic editors and reviewers

**Respected**

Onikepe Oluwa Damilola Owolabi

We would like to thank for the opportunity we get again to submit the revised version of the manuscript entitled “**What Determines Client Satisfaction on Labor and Delivery Service in Ethiopia**: Systematic Review and Meta-Analysis” to publish on Plose one. We appreciate you and the reviewer for the time and effort dedicated to review and give feedback on the manuscript. We critically observed the comments by the academic editors and reviewers, and we observed that the majority of the comments and concerns were addressed on the previous revisions and now we gave much emphasis on grammar and spelling errors which was raised by the reviews. So that we have tried to incorporate all the suggestions given by the academic editors and reviewers and we have made revision of the manuscript on track change. The point-by-point response to the reviewers’ concerns and comments are presented here below.

Point by point response for the comments from the academic editors and reviewer on manuscript entitled “**What Determines Client Satisfaction on Labor and Delivery Service in Ethiopia**: Systematic Review and Meta-Analysis”

1. **Reviewer's Responses to Questions**

**Comments to the Author**

If the authors have adequately addressed your comments raised in a previous round of review and you feel that this manuscript is now acceptable for publication, you may indicate that here to bypass the “Comments to the Author” section, enter your conflict-of-interest statement in the “Confidential to Editor” section, and submit your "Accept" recommendation.

**Reviewer #1: (No Response)**

**Authors response**

- We appreciate the feedback from of our academic editors and reviewers, and we incorporate all the points raised by our reviewers.

2. **Reviewer's Responses to Questions**

**Comments to the Author**

**Is the manuscript technically sound, and do the data support the conclusions?**
The manuscript must describe a technically sound piece of scientific research with data that supports the conclusions. Experiments must have been conducted rigorously, with appropriate controls, replication, and sample sizes. The conclusions must be drawn appropriately based on the data presented.

**Reviewer #1: Yes**

**Authors response**

- We saw the response and thankfull

3. **Reviewer's Responses to Questions**

**Comments to the Author**

**Has the statistical analysis been performed appropriately and rigorously?**

**Reviewer #1: Yes**

**Authors response**

- We saw the response and thankfull

4. **Reviewer's Responses to Questions**

**Comments to the Author**

**Have the authors made all data underlying the findings in their manuscript fully available?**
The PLOS Data policy requires authors to make all data underlying the findings described in their manuscript fully available without restriction, with rare exception (please refer to the Data Availability Statement in the manuscript PDF file). The data should be provided as part of the manuscript or its supporting information, or deposited to a public repository. For example, in addition to summary statistics, the data points behind means, medians and variance measures should be available. If there are restrictions on publicly sharing data—e.g. participant privacy or use of data from a third party—those must be specified.

**Reviewer #1: Yes**

**Authors response**

- We saw the response and thankfull

5. **Reviewer's Responses to Questions**

**Comments to the Author**

**Is the manuscript presented in an intelligible fashion and written in standard English?**
PLOS ONE does not copyedit accepted manuscripts, so the language in submitted articles must be clear, correct, and unambiguous. Any typographical or grammatical errors should be corrected at revision, so please note any specific errors here.

**Reviewer #1: No**

**Authors response to the academic editors and reviewer**

- We accepted the comment and inccorporated all the possible corections.
- **Review Comments to the Authors**

**Reviewer #1**: I think this is an important manuscript which should be published, and I believe the authors have put a lot of work and effort into the document. However there are grammatical and spelling mistakes, sometimes words - verbs - are missing in sentences.
I would It would be unfortunate if the manuscripot on this very important topic could not be published because of these reasons.I would therefore urge the authors to go through the manuscript again and check it thoroughly for spelling and grammatical mistakes. Please state what acronymns mean the first time you use them.Perhaps you can identify a native English speaker to help you?

**Authors response**

- We postively accepted the comment and invited two nativespeakers for gramer and editorial task and inccorporated all the possible corections.
